# The *miR-221/222* cluster, *miR-10b* and *miR-92a* are highly upregulated in metastatic minimally invasive follicular thyroid carcinoma

**DOI:** 10.3892/ijo.2013.1879

**Published:** 2013-04-02

**Authors:** TOMOO JIKUZONO, MASASHI KAWAMOTO, HIROSHI YOSHITAKE, KUNIO KIKUCHI, HARUKI AKASU, HITOSHI ISHIKAWA, MITSUYOSHI HIROKAWA, AKIRA MIYAUCHI, SHINICHI TSUCHIYA, KAZUO SHIMIZU, TOSHIHIRO TAKIZAWA

**Affiliations:** 1 Departments of Molecular Medicine and Anatomy, Nippon Medical School;; 2 Surgery, Division of Endocrine Surgery, Nippon Medical School;; 3 Division of Diagnostic Pathology, Nippon Medical School Hospital;; 4 Department of Clinical Pathology, University Hospital, Mizonokuchi, Teikyo University School of Medicine, Tokyo;; 5 Division of Endocrine Surgery, Nippon Medical School Musashi-kosugi Hospital, Kanagawa;; 6 Yamagata Saisei Hospital, Yamagata;; 7 Kuma Hospital, Hyogo, Japan

**Keywords:** microRNA, minimally invasive follicular thyroid carcinoma, thyroid surgery, metastasis, prognostic factor

## Abstract

Minimally invasive follicular thyroid carcinoma (MI-FTC) is characterized by limited capsular and/or vascular invasion with good long-term outcomes. However, some cases of MI-FTC show a poor prognosis because of severe distant metastasis (i.e., metastatic MI-FTC). Nonetheless, no method has been established for predicting the prognosis of MI-FTC. This study was conducted to identify novel prognostic factors for metastatic MI-FTC by the use of microRNA (miRNA). Thirty-four patients with MI-FTC were categorized into two groups: the metastatic group, M(+) (n=12) and the non-metastatic group, M(−) (n=22). In the M(+) group, distant metastasis was recognized after the initial operation established the diagnosis of MI-FTC. In the M(−) group, no distant metastasis was recognized postoperatively for ≥10 years. Using laser micro-dissection followed by quantitative real-time PCR and PCR arrays, we performed a comprehensive expression profiling of 667 miRNAs in formalin-fixed, paraffin-embedded samples from the initial MI-FTC operation. Furthermore, we assessed the potential use of miRNAs as novel biomarkers for the metastatic potential of MI-FTC by logistic regression analysis. Comprehensive quantitative analysis of miRNA expression in MI-FTC samples revealed that the *miR-221/222* cluster (i.e., *miR-221*, *miR-222* and miR-222^*^), *miR-10b* and *miR-92a* were significantly upregulated in the M(+) group compared with the M(−) group. Interestingly, the expression levels of these miRNAs were also shown to be upregulated in widely invasive FTC (WI-FTC; n=13) that has distant metastasis and worse prognosis, indicating a close similarity in the miRNA expression between metastatic MI-FTC and WI-FTC. Logistic regression analysis revealed that *miR-10b* made a significant contribution to prognosis (OR 19.759, 95% CI 1.433–272.355, p= 0.026). Our findings suggest that *miR-10b* is a potential prognostic factor for evaluating the metastatic potential of MI-FTC at an initial operation stage.

## Introduction

Follicular thyroid carcinoma (FTC) and papillary thyroid carcinoma (PTC) are major histological types of thyroid carcinoma that account for 10–20 and 75–85% of thyroid carcinomas, respectively (1–3). They are both classified as differentiated thyroid carcinomas originating from a common cell type (i.e., follicular cells) (4). In Japan, according to a nationwide cancer registry by the Japanese Society of Thyroid Surgery, a total of 52,109 patients with thyroid carcinoma underwent surgery between 1977 and 2005, including 4,910 (9.4%) cases of FTC and 45,683 (87.7%) cases of PTC [Saikawa *et al*, Abstracts of the 40th Annual Meeting of the Japanese Society of Thyroid Surgery, Japanese Society of Thyroid Surgery: pp121-136, 2007 (in Japanese)].

The pathological diagnosis and classification of FTC were based on the recent World Health Organization classification system (4). FTC is defined by the presence of vascular and/ or capsular invasion and the absence of diagnostic nuclear features of papillary carcinoma (4). This carcinoma is further divided into minimally invasive FTC (MI-FTC) and widely invasive FTC (WI-FTC) (4,5). MI-FTC has limited capsular and/or vascular invasion, whereas WI-FTC shows widespread infiltration of adjacent thyroid tissue and/or blood vessels. For tumors suspected of being MI-FTC, a standard operation method is thyroid lobectomy (www.endocrineweb.com/conditions/thyroid/thyroid-operations; accessed May 10, 2011) (5). MI-FTC shows good long-term outcomes. However, in some cases, MI-FTC metastasizes to the lung and bone, exhibiting a poor prognosis (i.e., metastatic MI-FTC). Nonetheless, distinguishing between metastatic and non-metastatic MI-FTCs is currently difficult by any pathological modalities. When distant metastasis is recognized during the follow-up period, additional therapies, such as completion total thyroidectomy and radioiodine ablation therapy, are needed (5). Thus, identification of prognostic biomarkers for predicting groups at high risk for metastasis among patients diagnosed with MI-FTC after the initial operation should be important in the postoperative follow-up of MI-FTC.

MicroRNAs (miRNAs) are endogenous, non-coding, small RNAs of 19–23 nucleotides in length that posttranscriptionally regulate the expression of their target genes at mRNA and/ or protein levels (6). So far, several miRNA profiling studies have demonstrated dysregulated miRNA expression in various types of human carcinomas and the potential use of miRNAs as diagnostic and/or prognostic markers was recently described (7–11). In terms of thyroid carcinomas, several reports have addressed dysregulated miRNA expression in PTC (12–14). On the other hand, information on miRNA expression in FTC, especially MI-FTC, is quite limited. In this study, we aimed to identify novel prognostic factors for metastatic MI-FTC and performed comprehensive profiling of miRNA expression in formalin-fixed, paraffin-embedded (FFPE) samples of FTC obtained at the initial operation using a combination of laser microdissection (LMD) and quantitative PCR-based array. Furthermore, we assessed the potential use of miRNAs as novel biomarkers for the metastatic potential of MI-FTC by logistic regression analysis.

## Materials and methods

### Patients and specimens

The records of 34 patients with MI-FTC who underwent surgery at Kuma Hospital (Hyogo, Japan) and Nippon Medical School Hospital (Tokyo, Japan) were selected from our archives of around 200 patients with MI-FTC between 1991 and 2009, of which the proportion of all thyroid cancers was 2–3%. The 34 cases met the following criteria: i) histopathological evaluation of the primary surgical specimens as MI-FTC was done according to the criteria of World Health Organization (4), ii) patients had undergone Tg (thyroglobulin) testing and neck ultrasonography routinely for ≥10 years after surgery and iii) patients were Tg antibodies-negative. This study was carried out in accordance with the principles embodied in the 1975 Declaration of Helsinki and informed consent for the use of thyroid tissues was obtained from each patient. We categorized 34 patients with MI-FTC into two groups: the metastatic group, M(+) (n=12) and the non-metastatic group, M(−) (n=22). In the M(+) group, distant metastasis was recognized after the initial operation established the diagnosis of MI-FTC. In the M(−) group, no distant metastasis was recognized postoperatively for ≥10 years. Although patients in both groups were clinicopathologically diagnosed with MI-FTC at the time of the initial operation, neither routine pathological examination nor clinical data could distinguish between the M(+) and M(−) groups. Clinical characteristics of each individual case are presented in Table I.

In order to further elucidate the miRNA expression profile characteristics of metastatic MI-FTC, we also analyzed the samples of patients with WI-FTC. All the records of patients with WI-FTC who underwent surgery at Kuma Hospital between 1998 and 2009 were collected (n=13). They met the criterion that histopathological evaluation of the primary surgical specimens as WI-FTC was done according to the criteria of World Health Organization (4); clinical characteristics of each individual case are presented in Table II.

### RNA purification from FFPE samples by LMD

The 34 archival FFPE samples of MI-FTC and 13 of WI-FTC were processed into 20-*μ*m sections and subjected to hematoxylin-eosin staining. We then microdissected areas containing carcinoma tissues in each section using an LMD microscope (LMD6000 System, Leica, Wetzlar, Germany).

The microdissected tissues were treated with xylene to remove paraffin and digested in a buffer containing 10% sodium dodecyl sulfate (Sigma-Aldrich, St. Louis, MO) and 20 mg/ml proteinase K (Roche Diagnostics, Mannheim, Germany) at 55°C with continuous stirring for 12 h. Total RNAs in these tissues were then extracted using Isogen-LS reagent (Wako, Osaka, Japan) according to the manufacturer’s protocol.

### Comprehensive quantitative analysis of miRNA expression using quantitative PCR-based array

Comprehensive analysis of miRNA expression levels in MI-FTC was performed by real-time PCR using TaqMan MicroRNA Array Panels (Applied Biosystems, Foster City, CA), which are designed to detect 667 human miRNAs. Equal quantities of total RNA isolated from each of 9 M(+) and 10 M(−) MI-FTC FFPE samples were pooled within the carcinoma groups. The pooled total RNAs (252 ng) were reverse-transcribed using Megaplex RT Primers (Applied Biosystems). These cDNAs were pre-amplified using Megaplex PreAmp Primers (Applied Biosystems). The pre-amplified products were applied to real-time PCR using TaqMan MicroRNA Assays Human Panels (A and B, v2.0) on a 7900HT Fast Real-Time PCR system (Applied Biosystems) according to the manufacturer’s instructions; miRNA sequences were annotated by the Sanger Data Base (miRBase) Release 14. Data obtained with this assay were analyzed using RQ Manager 1.2 (Applied Biosystems). For the quantification of each miRNA expression level, the relative Ct method (ΔΔCt method) was applied. *Small endogenous nucleolar RNA U44* (*RNU44*) was used as an internal control for data normalization.

### Quantitative analysis of miRNA expression by real-time PCR

Expression of individual miRNAs was validated using TaqMan miRNA assays (Applied Biosystems). Briefly, 10 ng total RNA was reverse-transcribed using a reverse transcription (RT) primer specific for individual miRNAs with MultiScribe Reverse Transcriptase (Applied Biosystems). The RT products were subsequently subjected to a PCR reaction with primer sets specific for individual miRNAs. Amplification of miRNA-derived PCR products was monitored on an ABI 7300 Real-Time PCR system (Applied Biosystems). All reactions were performed in triplicate and *RNU44* was used as a reference for data normalization. For absolute quantification of the expression levels of miRNAs, serially diluted synthetic mimics of these miRNAs and *RNU44* (Gene Design, Osaka, Japan) were used as standards.

### Statistical analysis

The statistical differences of miRNA expression among different groups [i.e., M(+) and M(−) MI-FTC groups and WI-FTC group] were analyzed by Kruskal-Wallis test.

As mentioned above, 9 M(+) and 10 M(−) MI-FTC FFPE samples were used for comprehensive analysis of miRNA expression levels in MI-FTC by PCR-based array. These training samples were later merged into the validation samples using the validation of miRNA expression in individual MI-FTC samples since it was difficult to collect further, more testing samples. Leave-one-out cross-validation was performed to protect overfitting and test the stability and predictive capability of our model using the entire 34 samples with MI-FTC. The overall predictive accuracy of the discriminant function, i.e., hit ratio was calculated. The classification accuracy was considered high when the hit ratio was calculated to be ≥25% greater than that achieved by chance (15).

To assess the prognostic value of miRNAs in the prediction of metastasis after the initial MI-FTC operation, odds ratios (ORs) with 95% confidence intervals (CIs) were calculated. Either the χ^2^ test or the Mann-Whitney U test was used to examine a possible association between metastatic status and clinicopathological parameters including miRNAs. Only variables that were significant in univariate analyses were used in a multivariate model. Multicollinearity was also assessed by using the variance inflation factor (VIF); a VIF exceeding 10 was regarded as indicating serious multicollinearity (16). Forced-entry binary logistic regression was used to predict the metastasis after the initial MI-FTC operation. We conducted all analyses using a statistical software package (SPSS for Windows, version 20, IBM-SPSS, Chicago, IL) and p-values <0.05 were considered statistically significant.

## Results

### Identification of miRNAs upregulated in FFPE samples of metastatic MI-FTC using a combination method of LMD and quantitative PCR-based miRNA expression array

To identify miRNAs with aberrant expression in metastatic MI-FTCs, we performed the initial experiments of comparison of miRNA expression profiles between 9 M(+) and 10 M(−) MI-FTC LMD FFPE samples using a real-time PCR-based miRNA expression profiling array (Table I; nos. 1–9 and 13–22). The pooled samples (equal amounts of RNA from each individual samples) were analyzed by real-time PCR-based array as an initial screening since the amounts of total RNAs extracted from LMD samples were limited. Considering the clinical use of miRNAs as potential biomarkers, those expressed at high levels in MI-FTCs should be advantageous in terms of sensitivity and reliability. Thus, we first screened miRNAs based on the Ct values, which were considered to roughly reflect the expression levels of these miRNAs. We preliminarily examined the expression levels of some miRNAs with Ct values >25 in the array analysis and found that in many, if not most, cases, the miRNAs were expressed at low levels, i.e., these Ct values were >35 or undetermined (17). It can be explained by the fact that cDNAs were pre-amplified for the array analysis. Thus, miRNAs with Ct values ≤25 in either M(+) or M(−) groups were subjected to further analysis; 178 miRNAs satisfied this criterion for all samples (Table III; the full data set is available upon request).

In the 178 miRNAs that met the above criteria, we then focused on the miRNAs that were upregulated or downregulated by >4.0-fold in the M(+) group compared to the M(−) group since the amounts of total RNAs extracted from LMD samples were limited. Six miRNAs, i.e., *miR-221*, *miR-222*, miR-222^*^, *miR-10b*, *miR-92a* and *miR-375*, were upregulated. Their expression levels were upregulated >4-fold in the M(+) group compared to the M(−) group (Table III). Three miRNAs, i.e., *miR-221*, *miR-222* and miR-222^*^, belongs to the *miR-221/222* cluster. Two miRNAs, i.e., *miR-888* and *miR-891a*, were downregulated >4-fold in the M(+) group compared to the M(−) group; the fold changes for *miR-888* and *miR-891a* were 0.02 and 0.03, respectively.

### Validation of miRNA expression in individual FTC samples by quantitative PCR

For the PCR-based array analysis described above, we used pooled RNA samples from the FTC samples; thus, only the averaged miRNA expression profiles could be obtained. For the validation of miRNA expression in individual MI-FTC samples, the cases of patients with MI-FTC were increased from 19 to 34 [12 and 22 samples for M(+) group and M(−) group, respectively]. However, due to the limited sample size, cross validation was used to protect overfitting and test the stability and predictive capability of our model using the entire 34 samples with MI-FTC. Clinical characteristics of these cases are summarized in Table IV. As seen in Table IV, only variables that were significant in univariate analyses were used for the cross-validation. Prognostic variables were age (continuous), vascular invasion (dichotomous) and four miRNAs (continuous; *miR-221*, miR-222^*^, *miR-10b* and *miR-92a*). The hit ratio was 76.5%; the cross validated classification showed that overall 76.5% were correctly classified.

**Figure 1:**
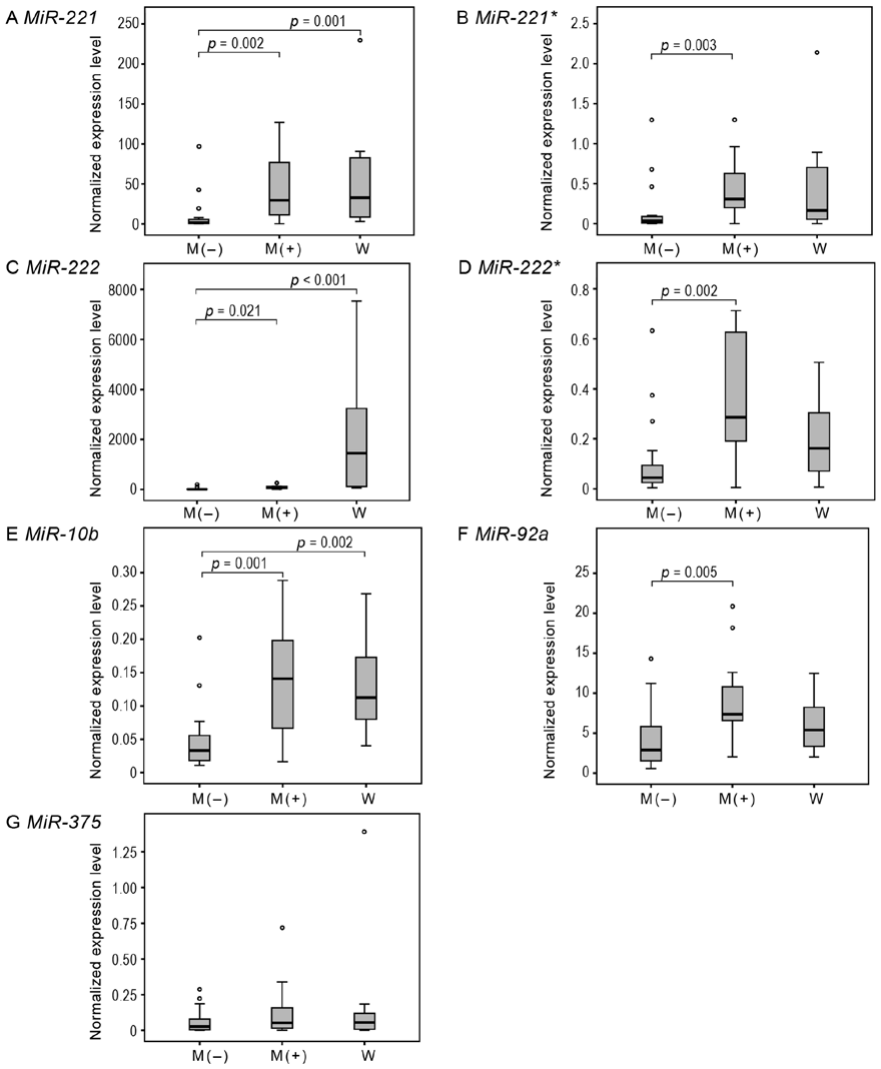
Quantitative PCR analysis to assess the expression levels of miR-221, miR-221^*^, miR-222, miR-222^*^, miR-10b, miR-92a and *miR-375* in MI-FTC and WI-FTC. Box plots show the expression levels of these miRNAs in M(+) MI-FTC and WI-FTC (W) samples compared to those in M(−) MI-FTC samples. The expression levels of these miRNAs were absolutely quantified and normalized for the expression level of *RNU44* in each sample; miRNA (amol/*μ*l)/*RNU44* (amol/*μ*l). Six miRNAs [(A) *miR-221*, (B) miR-221^*^, (C) *miR-222*, (D) miR-222^*^, (E) *miR-10b*, (F) *miR-92a* and (G) *miR-375*] are shown to be significantly upregulated in M(+) samples compared to M(−). Three miRNAs [(A) *miR-221*, (C) *miR-222* and (E) *miR-10b*] are shown to be significantly upregulated in W samples compared to M(−). Lines inside boxes denote medians, the boxes represent the interquartile range and whiskers extend to the most extreme values within 1.5 times the interquartile range. Outliers are indicated with circles. The statistical differences among these three groups are analyzed by Kruskal-Wallis test.

**Figure 2:**
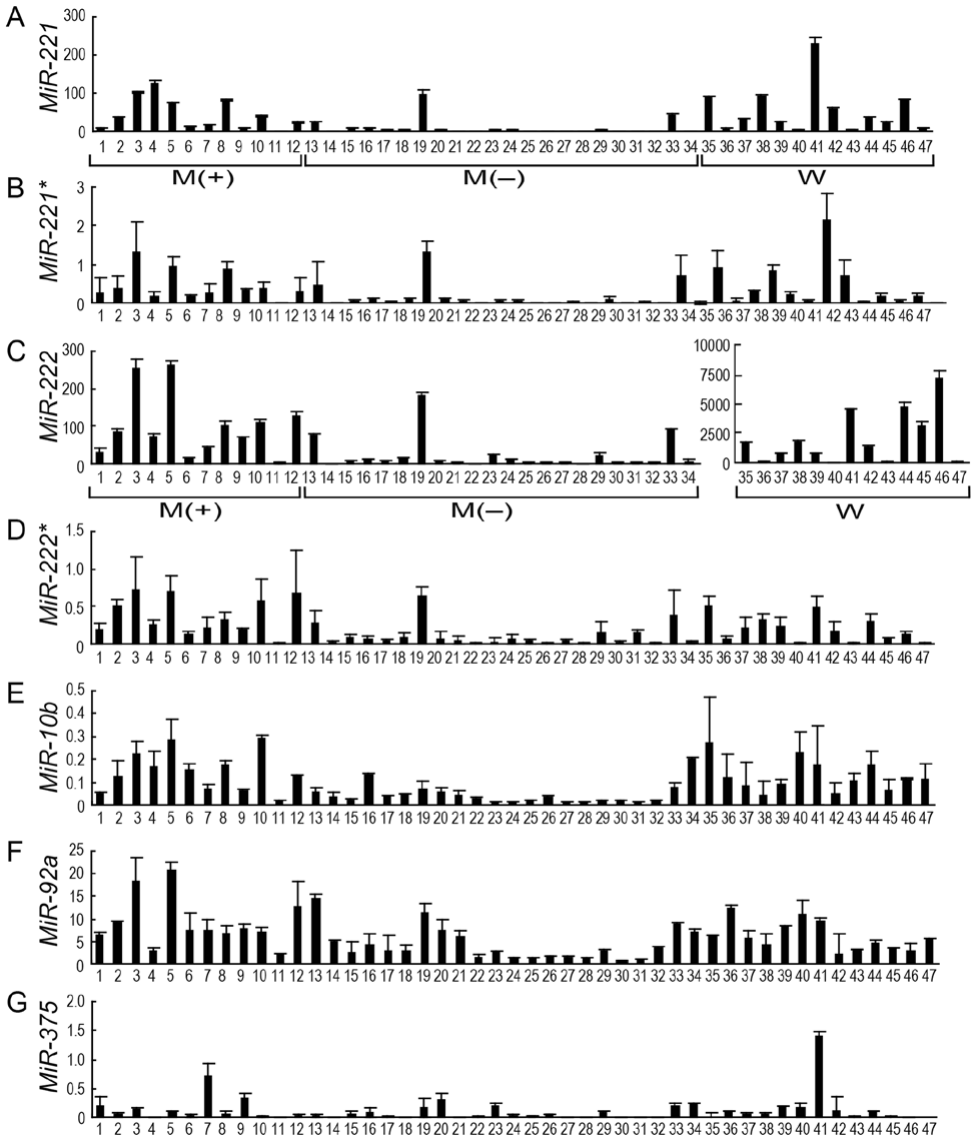
Histogram illustration showing the expression levels of miR-221, miR-221^*^, miR-222, miR-222^*^, miR-10b, miR-92a and *miR-375* in 47 samples of FTC. The expression levels of these miRNAs [(A) *miR-221*, (B) miR-221^*^, (C) *miR-222*, (D) miR-222^*^, (E) *miR-10b*, (F) *miR-92a* and (G) *miR-375*] in samples laser-microdissected from metastatic MI-FTC [M(+); nos. 1–12], non-metastatic MI-FTC [M(−); nos. 13–34] and WI-FTC (W; nos. 35–47). The expression levels of these miRNAs were absolutely quantified and shown as the values normalized for the expression level of *RNU44* in each sample; miRNA (amol/*μ*l)/*RNU44* (amol/*μ*l). The data are presented as the mean ± SD.

Then, we performed real-time PCR analysis to quantify and validate the expression of the miRNAs (Figs. 1 and 2). This miRNA expression analysis revealed that the expression levels of *miR-221*, *miR-222*, miR-222^*^, *miR-10b* and *miR-92a* were significantly upregulated in M(+) samples compared to M(−) samples (p=0.002, 0.021, 0.002, 0.001, 0.005, respectively), whereas, *miR-375* was not significantly upregulated in M(+) samples compared to M(−) samples (p=0.532). Note that another member of the *miR-221/222* cluster, miR-221^*^, was also shown to be significantly upregulated (p=0.003), although this miRNA did not meet the criterion for the screening based on our array analysis [the fold-change was 3.63, but Ct values of M(+) and M(−) were 27.12 and 28.98, respectively].

Furthermore, we investigated the expression levels of these miRNAs in WI-FTC samples to assess whether the miRNA expression patterns in metastatic MI-FTC were similar to those in WI-FTC that has distant metastasis and worse prognosis. Quantitative PCR analysis revealed that *miR-221*, *miR-222*, *miR-10b* and were significantly upregulated in WI-FTC tissues compared to M(−) tissues (p=0.001, p<0.001 and p=0.002, respectively) (Fig. 1). The p-values for miR-221^*^, miR-222^*^ and *miR-92a* were 0.070, 0.299 and 0.197, respectively. It should be noted that the expression pattern of upregulation of *miR-221/222* cluster and *miR-10b* in metastatic MI-FTC was substantially similar to that of WI-FTC (Fig. 1).

In addition, we also examined the expression levels of *miR-888* and *miR-891a*, in individual FTC samples by real-time PCR, these downregulated miRNAs were not significantly downregulated in M(+) samples compared to M(−) samples (data not shown).

### Evaluation of the prognostic values of miRNAs in MI-FTC

A logistic regression analysis was conducted to evaluate the prognostic values of miRNAs in the prediction of MI-FTC metastasis. As seen in Table IV, only variables that were significant in univariate analyses were used in a multivariate model. *MiR-222* was excluded as an independent variable since the value of VIF for *miR-222* was 18.332, indicating serious multicollinearity. The dependent variables were M(+) and M(−); independent variables considered in the model were age, vascular invasion and four miRNAs (*miR-221*, miR-222^*^, *miR-10b* and *miR-92a*). Forced-entry binary logistic regression was used to predict the metastasis after the initial MI-FTC operation. A test of the full model against a constant only model was statistically significant, indicating that the prognostic variables as a set reliably distinguished between M(+) and M(−) groups (χ^2^=25.552, p<0.001 with df=6). Prediction success overall was 85.3% [86.4% for M(−) and 83.3% for M(+)]. The Wald criterion demonstrated that only *miR-10b* made a significant contribution to prognosis [OR for a 1 standard deviation (SD)] increase 19.756, 95% CI 1.433–272.355, p=0.026). ORs, 95% CIs and p-values are summarized in Table V. These data imply that *miR-10b* has potential as a prognostic factor for MI-FTC.

## Discussion

In this study, we performed a comprehensive analysis of miRNA expression in MI-FTC and found that miRNAs comprising the *miR-221/222* cluster, *miR-10b* and *miR-92a* were significantly upregulated in metastatic MI-FTC. We used FFPE samples obtained from surgical operations for LMD analysis. Stocks of pathologic samples, such as FFPE specimens, are of great advantage in the design of clinical studies. Protein markers in FFPE tissues are generally stable provided that they are stored appropriately; thus, they can be detected by means of various immunohistological staining methods as well as mass spectrometry analysis. Conversely, RNAs are believed to be relatively unstable in FFPE samples and Xi *et al* indeed showed that the detectable levels of mRNA transcripts in freshly frozen samples and FFPE samples are poorly correlated (18). This could be explained in part by the fragmentation of mRNAs in FFPE samples; cellular RNAs were demonstrated to survive fixation and embedding procedures and RNA extraction as relatively short (<300-bp) fragments (19). Furthermore, RNAs in FFPE samples undergo some chemical modifications, which in turn facilitates fragmentation and interferes with enzymatic reactions such as RT (20,21). In contrast, small RNAs such as miRNAs have been demonstrated to be more stable, emerging as suitable molecules for the molecular characterization of FFPE samples (18). In addition, active miRNAs are present intracellularly as a complex with an RNA-induced silencing complex, which possibly protects miRNAs from degradation. Application of the LMD technique makes FFPE samples even more advantageous in selective regions, e.g., carcinoma regions. No prior study has been conducted using LMD for the molecular analysis of FTC. To our knowledge, this is the first study to achieve the comprehensive analysis of miRNA expression in MI-FTC using LMD.

The *miR-221/222* cluster consists of four miRNAs: *miR-221*, miR-221^*^, *miR-222* and miR-222^*^. *miR-221* and *miR-222* were also previously reported to be upregulated miRNAs in PTC (12–14). In addition, *miR-221* and *miR-222* were identified as upregulated in FTC (22). Thus, these miRNAs appear to be closely linked with the pathogenesis of both PTC and FTC. In contrast, miR-221^*^ and miR-222^*^ have been considered to be minor miRNAs and the expression of these miRNAs in thyroid tumors has not been well studied. Judging from the Ct values in our array analysis, however, miR-222^*^ was expressed at considerable levels in FTC and this miRNA, as well as those of *miR-221* and *miR-222*, were dysregulated in metastatic MI-FTC compared to non-metastatic MI-FTC (Table III). Takano *et al* screened differentially expressed mRNAs in FTC and follicular adenoma and found the decreased expression of *trefoil factor 3* mRNA to be a marker of FTCs (23). Foukakis *et al* generated the mRNA expression profiles by PCR-based quantification followed by logistic regression analysis and attempted to identify transcriptional markers of malignancy in FTC (24). However, no mRNAs that distinguish between metastatic and non-metastatic MI-FTCs have been reported so far. Interestingly, Lu *et al* reported that miRNA profiles are highly informative for the classification of poorly differentiated tumors, the classification of which was inaccurate by mRNA profiles (25). Likewise, our findings suggest that miRNA profiles allow us to classify MI-FTC into metastatic and non-metastatic groups, which have not been previously distinguished, in terms of molecular pathology. Molecular pathology examinations of miRNAs for the metastatic potency of MI-FTC using FFPE surgical samples from the initial operation should lead to recommendations for patients to strictly monitor metastatic signs at intervals and if necessary undergo additional operations (completion total thyroidectomy) and radioiodine ablation therapy.

What functions do these miRNAs have? Recent studies have reported that *miR-221* and *miR-222* regulate cell growth and cell cycle progression by targeting *cyclin-dependent kinase inhibitor 1B (CDKN1B)* and *cyclin-dependent kinase inhibitor 1C (CDKN1C)* in several cancer cell lines (26–30). This miRNA-mediated cell cycle regulation was also reported for PTC (12,31). Considering that both FTC and PTC are differentiated thyroid carcinomas originating from a common cell type (the follicular cell), the upregulation of *miR-221* and *miR-222* in MI-FTC may lead to dysregulated cell cycle progression by targeting *CDKN1B* and *CDKN1C* and facilitates its hematogenous metastasis to lung and bone. Another possibility is that the *miR-221* gene family is involved in metastatic processes by affecting cell migration and/or invasion. *In silico* target prediction analysis revealed that these miRNAs possibly target genes associated with matrix degradation. For example, TargetScan 5.1 (www.targetscan.org/; Accessed May 10, 2011), a representative target prediction program, predicts that both *miR-221* and *miR-222* putatively target the mRNAs for tissue inhibitor of metalloproteinase (TIMP)-2 and TIMP-3, endogenous inhibitors of metalloproteinases (MMPs) such as MMP-2 and -9. Because these MMPs degrade matrices, particularly basement membranes, to facilitate cancer cell invasion, *miR-221* and *miR-222* possibly enhance cancer metastasis by downregulating MMP inhibitors. *MiR-10b* has also been reported to have malignancy and metastatic behavior; *miR-10b* is upregulated in several cancer types (32–36). Ma *et al* revealed that *miR-10b* initiates tumor invasion and metastasis in cancer cells (32). In breast cancer cells, the upregulation of *miR-10b* suppresses a direct target *Homeobox D10 (HOXD10)*, leading to induction of a pro-metastatic gene, *ras homolog gene family, member C*(32). Sun *et al* demonstrated that in glioma cells, *miR-10b* promotes cancer invasion by modulating tumor invasion factors MMP-14 and uPAR expression via *HOXD10*(37). It is likely that *miR-10b* acts as a promoter of metastasis in breast cancer cells (38). Upregulation of *miR-92a*, a *miR-17-92a* cluster-derived miRNA, has also been reported in various cancers (39–45). Recent report suggests a mechanism by which *miR-92a* promotes metastasis (46). Considering these metastasis-related miRNAs were upregulated in FTCs containing metastatic MI-FTC and WI-FTC, the dysregulation of these miRNAs could be closely related to the molecular mechanisms of metastasis in FTCs. Further studies will be necessary to elucidate the functions of the *miR-221/222* cluster, *miR-10b* and *miR-92a* in MI-FTC, especially, involvement in the molecular pathogenesis of MI-FTC metastasis. In addition, controversy exists involving the diagnostic criteria of well-differentiated thyroid carcinomas (FTC and PTC). Mete and Asa recently reported that the application of rigid criteria of vascular invasion (exclusion of cases with vascular pseudoinvasion) provided a clinically relevant prediction of distant metastasis in patients with PTC (47). It also remains to be investigated whether the levels of miRNA expression correlate with angioinvasion in well-differentiated thyroid carcinomas re-evaluated by the new criteria based on true vascular invasion.

The most remarkable point of the present study is that it was designed as a retrospective study to identify miRNAs dysregulated in metastatic MI-FTC over non-metastatic MI-FTC, in which we managed to collect specimens from patients who underwent ≥10 years of follow-up after the initial operation. Note that such a comparative analysis has not been previously achieved because of the difficulty in collecting FFPE samples and information from patients with non-meta-static MI-FTC who have undergone postoperative follow-up for such a long period. Logistic regression analysis further supports the clinical significance of our findings for surgical therapy in MI-FTC. However, the present and previous studies on thyroid carcinoma constituted a relatively small proportion of metastatic MI-FTC (23,24,48,49) since metastatic MI-FTC is a relatively rare form of thyroid cancer. The number of metastatic MI-FTC cases (in the total number of MI-FTC cases) employed in the studies by Takano *et al*(23), Foukakis *et al*(24), Asari *et al*(48) and Sugino *et al*(49) was 1 case (15 cases), 4 cases (31 cases), 12 cases (127 cases) and 20 cases (111 cases), respectively. In the present study, the number of metastatic MI-FTC (in the total number of MI-FTC) is 12 out of 34 cases. It also remains to be investigated whether the dysregulated miRNAs serve as surrogate endpoint biomarkers for MI-FTC. Therefore, a large multi-center case-control study of MI-FTC with a longer follow-up period is necessary for evaluation the clinical significance of the miRNAs identified in this study.

If patients are treated curatively, FTC and PTC exhibit essentially identical 10-year cause-specific survival rates (5,50–52). In terms of diagnosis, PTC is readily diagnosed by fine-needle aspiration cytology (FNAC). Conversely, FTC, especially MI-FTC, is difficult to diagnose preoperatively by any modality, including FNAC, because its routine cytological features are similar to those of follicular adenoma (5,53). Ideally, the preoperative prediction of the metastatic prognosis of MI-FTC, which could be achieved by detecting miRNAs in FNAC samples, should be possible because the upregulation of *miR-221* and *miR-222* has been recently demonstrated in PTC from FNAC samples (14). However, further studies are needed regarding the clinical applications for MI-FTC.

In conclusion, our miRNA analysis in FFPE samples using LMD has provided important information regarding the molecular pathology and novel therapeutic strategies for MI-FTC. In this study, we found for the first time that the expression of miRNAs belonging to the *miR-221/222* cluster, *miR-10b* and *miR-92a* were significantly upregulated in meta-static MI-FTC. We conclude that *miR-10b* shows potential as a prognostic factor for MI-FTC at an initial operation stage.

## Figures and Tables

**Table I:** Clinical features of the MI-FTC patients in this study.

						Tg (ng/ml)^d^		Invasion	Distant metastasis after surgery		
		
No.	Metastasis	Sex	Age^a^	Tumor size (mm)^b^	Operation method^c^	Pre-operation	Post-operation	c/v^e^	Period (month)	Location	Additional therapies
1	+	M	67	49	Lo	18.9	unknown	+/+	85	B	TT and RAT
2^f^	+	M	68	75	TT	>8000	4.2	+/+	51	L, B	
3^g^	+	F	68	90	TT	>8000	130.0	+/+	36	L	
4	+	M	15	86	Lo	6987.0	17.0	+/+	26	L	TT and RAT
5	+	F	51	60	TT	4067.0	11.4	+/+	40	L	RAT
6	+	F	50	19	Lo	46.6	8.1	+/−	36	L, B	TT and RAT
7	+	F	58	38	Lo	179.0	10.1	+/−	72	L, B	TT and RAT
8	+	F	47	23	Lo	640.0	6.4	+/+	98	B	TT and RAT
9	+	M	72	48	Lo	770.0	17.0	+/+	62	L	TT and RAT
10	+	F	57	90	TT	>8000	162.6	−/+	13	B	RAT
11	+	M	54	58	Lo	83.3	25.3	+/+	68	L	TT and RAT
12	+	F	47	45	TT	3870	<0.5	+/−	26	B	RAT
13	−	M	53	45	Lo	2118.0	13.3	+/−		na	
14	−	F	49	45	Lo	814.0	<2.0	+/−		na	
15	−	F	55	15	Lo	51.7	17.9	+/−		na	
16	−	F	38	23	Lo	581.0	17.0	+/−		na	
17	−	F	63	65	Lo	1269.0	51.3	+/−		na	
18	−	F	64	46	Lo	2024.0	5.3	+/−		na	
19	−	F	50	28	Lo	75.7	24.8	+/+		na	
20	−	F	33	24	ST	399.0	<2.0	+/+		na	
21	−	F	37	47	Lo	81.2	3.6	+/−		na	
22	−	F	28	40	Lo	183.0	37.0	+/−		na	
23	−	F	37	55	Lo	1049.0	9.6	+/+		na	
24	−	F	47	57	Lo	1724.0	13.7	+/−		na	
25	−	M	74	32	Lo	50.2	19.2	+/+		na	
26	−	F	32	31	Lo	67.6	19.0	+/+		na	
27	−	M	64	49	Lo	2153.4	20.6	+/−		na	
28	−	M	35	30	Lo	401.1	10.0	+/−		na	
29	−	F	29	40	Lo	1049.2	9.5	+/−		na	
30	−	F	25	46	Lo	1051.9	7.0	+/−		na	
31	−	M	38	44	TT	343.4	<0.5	+/−		na	
32	−	F	23	33	Lo	78.6	10.8	+/−		na	
33	−	F	50	77	TT	4640	4.1	+/−		na	
34	−	M	29	78	Lo	178.4	23.1	+/−		na	

F, female; M, male; TT, total thyroidectomy; ST, subtotal thyroidectomy; Lo, right or left thyroid lobectomy; Tg, thyroglobulin (normal range 2.0–35.0 ng/ml); c, capsular invasion; v, vascular invasion; L, lung; B, bone; RAT, radioiodine ablation therapy; na, not applicable.

a Age, median 54.5 in M(+) and 43.3 in M(−) (range 15–74).

b Tumor size: median 56.8 and 43.2 (range 15–90).

c Twenty-four of the 34 patients underwent thyroid lobectomy based on the standard operation method. Five of the 12 patients in the M(+) group underwent total thyroidectomy at the time of the initial operation because of high Tg values; thus, they did not require an additional operation for the purpose of radioiodine ablation therapy.

d Thyroglobulin (Tg) is used as a post-operative marker for the follow-up of patients with thyroid carcinoma. The Tg values were abnormal before the operation, but were reduced to within the normal range after the operation in almost all cases.

e Invasion: +, present; -, absent.

f Costectomy was performed at another hospital.

g No additional therapies had performed because of patients’ will.

**Table II:** Clinical features of the WI-FTC patients in this study.

					Distant metastasis		
No.	Metastasis	Sex	Age^a^	Operation method	Period (month)	Location	Additional therapies
35	+	M	43	Lo	23	L	TT and RAT
36	+	F	75	TT	0	L, T	RAT
37	+	F	76	TT	0	L	RAT
38	+	F	60	TT	101	L	RAT
39	+	F	56	TT	54	B	RAT
40	+	F	34	TT	39	L	RAT
41	+	F	42	TT	93	L, B	RAT
42	+	F	63	Lo	51	L	TT and RAT
43	+	M	63	TT	0	L	RAT
44	+	F	65	TT	15	B	RAT
45^b^	−	F	39	Lo		na	
46^b^	−	F	37	Lo		na	
47^b^	−	F	36	Lo		na	

F, female; M, male; TT, total thyroidectomy; Lo, right or left thyroid lobectomy; L, lung; B, bone; T, tumor embolism; RAT, radioiodine ablation therapy; na, not applicable.

a Age: median 53.0 (range 34–76).

b The patients in number 45–47 are followed up in post-operation within 2 years.

**Table III:** Representative miRNAs highly upregulated in the metastatic MI-FTC, as revealed by quantitative PCR-based array.

		Ct value^b^	
miRNA^a^	Fold change	M(+)	M(−)
***miR-375***	12.34	20.34	23.97
***miR-222***	11.44	12.05	15.57
***miR-221***	6.36	17.72	20.39
***miR-10b***	5.04	19.64	21.97
miR-222^*^	4.99	20.65	22.97
***miR-92a***	4.01	19.14	21.14
*miR-16*	3.70	14.45	16.34
*miR-31*	3.69	18.08	19.96
*miR-29b*	3.69	21.08	22.96
miR-130b^*^	3.57	23.17	25.01
*miR-204*	3.50	19.16	20.97
*miR-181c*	3.43	22.21	23.99
*miR-296-5p*	3.39	22.15	23.91
*miR-26a*	3.39	16.22	17.98
*let-7c*	3.36	19.90	21.65
*miR-135a*	3.32	16.26	17.99
*miR-125a-5p*	3.31	19.23	20.96
*miR-23b*	3.22	20.48	22.17
*miR-146a*	3.19	17.30	18.97
*miR-130b*	3.15	22.32	23.98
*miR-328*	3.12	19.00	20.64
*miR-454*	3.11	18.43	20.07
*miR-106a*	3.10	16.32	17.95
*miR-130a*	3.10	19.79	21.42
*miR-17*	3.08	17.06	18.68
*miR-101*	3.03	23.35	24.95
*miR-320*	3.00	18.39	19.97
*miR-605*	3.00	23.10	24.68

a MiRNAs that were upregulated >3.0-fold in the M(+) group compared to the M(−) group; miRNAs that were upregulated >4.0-fold in the M(+) group compared to the M(−) group are indicated in bold.

b Ct values of miRNAs were normalized to *RNU44*.

**Table IV:** Summary of clinicopathological features of the MI-FTC patients in this study.

	M(+) n=12	M(−) n=22	P-value
Sex			
Female/male	7/5	16/6	0.315^e^
Age	54.5±15.2	43.3±14.4	0.028^f^
Tumor size (mm)	56.8±24.5	43.2±16.3	0.094^f^
Operation method			
Lo/ST+ TT	7/5	19/3	0.080^e^
Tg (ng/ml)^a^			
Pre-operation	3388.5±3511.0	926.5±1097.3	0.217^f^
Post-operation	32.8±54.0	14.6±12.1	0.986^f^
Invasion^b^			
Capsular invasion +/−	11/1	22/0	0.353^e^
Vascular invasion +/−	9/3	5/17	0.005^e^
Distant metastasis after surgery			
Period (month)	51.1±26.1	na	
Location			
Lung	8	na	
Bone	7		
Additional therapies^c^	10	na	
Expression level of miRNAs^d^			
*miR-221*	43.792±41.809	9.351±21.818	<0.001^f^
*miR-222*	97.800±84.473	22.455±42.031	<0.001^f^
miR-222^*^	0.372±0.250	0.102±0.150	<0.001^f^
*miR-10b*	0.149±0.088	0.045±0.045	<0.001^f^
*miR-92a*	9.032±5.597	4.137±3.637	<0.001^f^
*miR-375*	0.136±0.209	0.065±0.086	0.309^f^

Lo, right or left thyroid lobectomy; ST, subtotal thyroidectomy; TT, total thyroidectomy; na, not applicable.

a Thyroglobulin (Tg, normal range 2.0–35.0 ng/ml) is used as a post-operative marker for the follow-up of patients with thyroid carcinoma. The Tg values were abnormal before the operation, but were reduced to within the normal range after the operation in almost all cases.

b Invasion: +, present; −, absent.

c Additional therapies mean a combination of total thyroidectomy and radioiodine ablation therapy or radioiodine ablation therapy.

d The expression levels of these miRNAs were absolutely quantified and shown as the values normalized for the expression level of *RNU44* in each sample; miRNA (amol/*μ*l)/*RNU44* (amol/*μ*l).

e χ^2^ test.

f Mann-Whitney U test; the results are expressed as mean ± SD.

**Table V:** Prognostic factors for prediction of MI-FTC metastasis.

Prognostic factor	P-value	OR (per SD increase)	95% CI
*miR-10b*	0.026	19.759	1.433–272.355
*miR-92a*	0.695	0.495	0.015–16.592
*miR-221*	0.706	0.674	0.087–5.232
miR-222^*^	0.508	2.960	0.119–73.725
Vascular invasion	0.110	12.650	0.564–283.580
Age	0.129	1.091	0.975–1.220
Constant	0.045	0.002	
